# Video-Rate Bioluminescence Imaging of Degranulation of Mast Cells Attached to the Extracellular Matrix

**DOI:** 10.3389/fcell.2018.00074

**Published:** 2018-07-10

**Authors:** Satoru Yokawa, Takahiro Suzuki, Ayumi Hayashi, Satoshi Inouye, Yoshikazu Inoh, Tadahide Furuno

**Affiliations:** ^1^School of Pharmacy, Aichi Gakuin University, Nagoya, Japan; ^2^School of Dentistry, Aichi Gakuin University, Nagoya, Japan; ^3^Yokohama Research Center, JNC Corporation, Yokohama, Japan

**Keywords:** bioluminescence, imaging, degranulation, mast cell, extracellular matrix

## Abstract

Degranulation refers to the secretion of inflammatory mediators, such as histamine, serotonin, and proteases, that are stored within the granules of mast cells and that trigger allergic reactions. The amount of these released mediators has been measured biochemically using cell mass. To investigate degranulation in living single cells, fluorescence microscopy has traditionally been used to observe the disappearance of granules and the appearance of these discharged granules within the plasma membrane by membrane fusion and the movement of granules inside the cells. Here, we developed a method of video-rate bioluminescence imaging to directly detect degranulation from a single mast cell by measuring luminescence activity derived from the enzymatic reaction between *Gaussia* luciferase (GLase) and its substrate coelenterazine. The neuropeptide Y (NPY), which was reported to colocalize with serotonin in the secretory granules, fused to GLase (NPY-GLase) was efficiently expressed in rat basophilic leukemia (RBL-2H3) cells, a mast-cell line, using a preferred human codon-optimized gene. Bioluminescence imaging analysis of RBL-2H3 cells expressing NPY-GLase and adhered on a glass-bottomed dish showed that the luminescence signals from the resting cells were negligible, while the luminescence signals of the secreted NPY-GLase were repeatedly detected after the addition of an antigen. In addition, this imaging method was applicable for observing degranulation in RBL-2H3 cells that adhered to the extracellular matrix (ECM). These results indicated that video-rate bioluminescence imaging using GLase will be a useful tool for detecting degranulation in single mast cells adhered to a variety of ECM proteins.

## Introduction

Mast cells secrete prestored allergic mediators (e.g., histamine and serotonin), synthesize lipid mediators (e.g., leukotorienes and prostaglandins), and produce cytokines (e.g., tumor necrosis factor-α and interleukin-4). These cells are important effectors of immediate allergic responses (Kalesnikoff and Galli, 2008) and are distributed throughout a variety of tissues and organs, some of which are influenced by mechanical stretch, such as heartbeat and respiration, and adhere to extracellular matrix (ECM) proteins, such as fibronectin, vitronectin, and laminin, by binding to their adhesion molecules, including integrin (Columbo et al., 1995; Kraft et al., 2005). Adhesion of mast cells to ECM proteins is known to enhance cell degranulation by crosslinking to high-affinity immunoglobulin E (IgE) receptors (FcεRI) on their plasma membrane (Hamawy et al., 1992). In turn, the activation of these mast cells amplifies their adhesion to ECM proteins (Thompson et al., 1990).

Traditionally, mast cell degranulation has been biochemically analyzed by measuring the amount of histamine or β-hexosaminidase secreted from a mass of cells; however, both electron microscopy and fluorescence microscopy have been used to observe degranulation at the single-cell level. In addition, a transmission electron microscope and a scanning electron microscope have been used to observe the discharged granules and ruffling of the plasma membrane after degranulation, respectively (Röhlich et al., 1971; Pfeiffer et al., 1985). An atomic force microscope was also used to observe the morphological changes in mast cells during the degranulation process (Nakamura and Nakanishi, 1999, 2000), and several methods have been examined for detecting degranulation in living mast cells using fluorescence microscopy. One such method is to simply stain the intracellular granules with a basic fluorescent probe, such as quinacrine and acridine orange (Kawasaki et al., 1991; Tamura et al., 2006); however, this stain is not highly selective because it also accumulates in acidic organelles. Another simple method is to label the marker proteins inside the granules (e.g., β-hexosaminidase and neuropeptide Y [NPY]) or in the granule membrane (e.g., CD63 and VAMP8) with fluorescent proteins (Amano et al., 2001; Nishida et al., 2005; Azouz et al., 2012, 2014; Wilson et al., 2016). Fluorescence microscopy is then a suitable method by which to analyze the movement of intracellular granules. However, it does not necessarily help to quantify the amount of proteins secreted from individual cells because this exocytotic event is generally detected as the disappearance of fluorescence intensity after the fluorescence probe is released to outside the cell. To address this using a different method, pH-sensitive fluorescent reporter proteins (pHluorins) were adopted to detect degranulation (Miesenböck et al., 1998; Horiguchi et al., 2016). Because the fluorescent characteristic of these reporter proteins changes according to environmental pH, degranulation can be detected by an increase in fluorescence intensity. The fluorescent avidin, which binds to negatively charged proteoglycans in the granules, was also used to detect the discharged granules on the cell surface (Joulia et al., 2015). Using these methods, fluorescence signals are accumulated on the plasma membrane in accordance with the degranulation process; however, quantification and specificity to detect degranulation remain restricted.

In our study, we used video-rate bioluminescence microscopy to directly visualize exocytosis in living mast cells. A method of bioluminescence imaging has been developing to both spatiotemporally and quantitatively study the secretion of proteins and peptide hormones from the entire surface of a cell (Inouye et al., 1992; Suzuki et al., 2007, 2011a,b; Suzuki and Inouye, 2014). By using secretory *Gaussia* luciferase (GLase) as a reporter protein, the luminescence signals of GLase during exocytosis were visualized at a video rate of 30–500 ms/frame using an electron-multiplying charge-coupled device (EM-CCD) camera. GLase is a small luciferase (16.8 kDa without the signal peptide sequence) and displays high luminescence on its expression in the endoplasmic reticulum (ER)–Golgi secretory pathway (Tannous et al., 2005). In a previous study, we successfully visualized glucagon and insulin secretions from pancreatic α and β cells using stimulation (Suzuki et al., 2011a; Yokawa et al., 2017). Both depolarization-induced glucagon secretion and glucose-stimulated insulin secretion were frequently observed at the intercellular contact regions of clustered α and β cells, respectively. In addition, oscillated and synchronized insulin secretion were visualized in ~100-μm-thick islets and spheroids of 3D-cultured β cells in response to stimulation with high concentration of glucose (Suzuki et al., 2017). As with pancreatic islet cells, we attempted to visualize exocytosis in mast cells stimulated with an antigen in real time. We consider that this technique could be developed to analyze the exocytotic events in single mast cells that are adhered to ECM proteins on thick gels.

## Materials and methods

### Plasmids

To express GLase in mast cells, we used two expression vectors—pcDNA3-hGLuc for the human codon-optimized GLase gene (*hGLuc*) (Tannous et al., 2005), and pcDNA3-pGLuc for the preferred human codon-optimized *GLuc* (*pGLuc*)—that contain the signal peptide sequence for secretion under the control of the cytomegalovirus (CMV) promoter (Inouye et al., 2015; Inouye and Suzuki, 2016). The preferred human codon-optimized gene of NPY (*pNPY*) was designed as previously described using a human codon usage table (http://www.kazusa.or.jp/codon/) based on the wild-type gene for human NPY with the signal peptide sequence (NCBI Reference Sequence: NP_000896.1) (Supplementary Figure 1A) (Inouye and Suzuki, 2016; Yokawa et al., 2017). The synthetic gene *pNPY* with the Kozak consensus sequence was obtained from Eurofins Genomics (Tokyo, Japan). To express the NPY protein fused to the amino terminus of GLase (NPY-GLase), the *Hin*dIII-*Eco*RI fragment of *pNPY* was inserted into pcDNA3-pGLuc-pN (Yokawa et al., 2017) to obtain pcDNA3-pNPY-pGLuc (Supplementary Figure 1B).

### Cell culture and transfection

The rat mast cell line RBL-2H3, the most widely used as a model for mast cells, was cultured in minimum essential medium (Nissui, Tokyo, Japan) supplemented with 10% fetal calf serum (Roche, Mannheim, Germany) at 37°C in a humidified atmosphere containing 5% CO_2_. To express GLase and NPY-GLase, the RBL-2H3 cells were electroporated with plasmids using Nucleofector II and Nucleofector Solution T (Lonza, Basel, Switzerland) (Furuno et al., 2015). To establish a stable transformant of the RBL-2H3 cell line expressing NPY-GLase, the cells were transfected with pcDNA3-pNPY-pGLuc and selected in G418 (0.5 mg/mL) (Sigma-Aldrich, St. Louis, MO, USA). A clonal transformant with highest luminescence activity and responsiveness to an antigen was obtained and was assigned as “RBL-NPY-GLase cells” (clone #14).

### β-hexosaminidase secretion

We monitored RBL-2H3 degranulation by measuring the activity of the granule-stored enzyme β-hexosaminidase that was secreted into the cell supernatants. Cells were plated onto 24-well plates at 5 × 10^4^ cells/well. The following day, cells were sensitized with anti-dinitrophenyl (DNP) IgE (1:5,000) at 37°C for 30 min, and the monolayers washed with 4-(2-hydroxyethyl)-1-piperazineethanesulfonic acid (Hepes) buffer (10 mM Hepes [pH 7.2], 140 mM NaCl, 5 mM KCl, 1 mM CaCl_2_, 0.6 mM MgCl_2_, 0.1% glucose, 0.1% bovine serum albumin [BSA], and 0.01% sulfinpyrazone) and incubated with DNP-conjugated BSA (DNP-BSA; 50 and 500 ng/mL) at 37°C. Following incubation, the supernatant aliquots were transferred to 96-well plates (20 μL/well) and incubated with 20 μL substrate solution (2 mM *p*-nitrophenyl-N-acetyl-β-D-glucosaminide (Sigma-Aldrich, St. Louis, MO, USA) in 100 mM citrate buffer [pH 4.5]) at 37°C for 1 h. After terminating the reaction using 167 mM Na_2_CO_3_-NaHCO_3_ buffer (160 μL), the absorbance at 405 nm was measured using a microplate reader (model 680, Bio-Rad, Hercules, CA, USA) (Furuno et al., 2015; Inoh et al., 2017). Release activity relative to total cellular β-hexosaminidase content was determined by disrupting the cells with × 1 passive lysis buffer (Promega, Fitchburg, WI, USA). Each assay was performed in triplicate.

### Measurement of luminescence activity of GLase and NPY-GLase using a luminometer

To compare the expression levels of GLase and NPY-GLase in RBL-2H3 cells using the various expression vectors, RBL-2H3 cells (5 × 10^4^ cells/well) cultured for 24 h in a 24-well plate were transfected with 0.5 μg of the expression vectors for GLase or NPY-GLase. After incubation, the culture medium was collected by centrifugation at 300 × g at 4°C for 5 min to remove the detached cells. To the culture medium (1 μL) were added 5 μg/mL coelenterazine (50 μL; JNC Co. Ltd., Tokyo, Japan) to determine the luminescence activity of GLase. The initial maximum light intensity was measured using an Atto AB2200 luminometer (ver. 2.61D; Atto Corp., Tokyo, Japan) equipped with an R4220P photomultiplier (Hamamatsu Photonics, Shizuoka, Japan) (Suzuki et al., 2017; Yokawa et al., 2017).

To analyze the NPY-GLase secreted from the RBL-NPY-GLase cells stimulated with DNP-BSA, the cells were plated onto 24-well plates at 5 × 10^4^ cells/well. The following day, the cells were sensitized with anti-DNP IgE (1:5,000) at 37°C for 30 min, and the monolayers washed with Hepes buffer and incubated with DNP-BSA (50 and 500 ng/mL) at 37°C for 30 min. After the supernatant was collected by centrifugation at 300 × g at 4°C for 5 min to remove the detached cells, the luminescence activity in the culture medium was measured as described above.

### Bioluminescence imaging

The methods of video-rate bioluminescence imaging were essentially the same as previously described (Suzuki et al., 2011a,b, 2017; Suzuki and Inouye, 2014; Yokawa et al., 2017). Bioluminescence signals were monitored at 37°C using the IX81-ZDC2 microscope (Olympus, Tokyo, Japan) equipped with a thermostat incubator (Tokai Hit, Shizuoka, Japan) and water-cooled EM-CCD camera (ImagEM 1K, model C9100-14, 1024 × 1024 pixels, pixel size = 13 μm; Hamamatsu Photonics, Shizuoka, Japan) in a light-proof box using a high numerical aperture objective lens (UPLSAPO 20 × dry, NA 0.75; Olympus, Tokyo, Japan). Data on the bioluminescence signals were recorded on a computer hard disk using AQUACOSMOS version 2.6 (Hamamatsu Photonics, Shizuoka, Japan) with an acquisition mode of 1 × 1 binning, fast scanning, electron-multiplying gain level 255, and photon imaging mode 1. The luminescence signals were acquired using an exposure time of 500 ms and a reading time of 1.712 ms/image. The luminescent video images were processed and analyzed using AQUACOSMOS. To analyze time-dependent changes in the amount of secreted reporter proteins, the average luminescence intensities within a confined area of a video image were calculated. To analyze the secretory luminescence signals localized on individual cells, a composite image (colored green) was generated that comprised maximum luminescence intensities from successive frames of a video image and was superimposed on the bright-field image.

For bioluminescence video imaging of secreted NPY-GLase in live cells, the RBL-NPY-GLase cells (5 × 10^5^ cells) cultured for 24 h on a 35-mm glass-bottom dish coated with or without Matrigel (BD Bioscience, Franklin Lakes, NJ, USA) were sensitized with anti-DNP IgE (1:5,000) at 37°C for 30 min and washed with Hepes buffer. The focus position of the z-axis was adjusted to 2 μm from the bottom side of the cells attached to the glass plate using the ZDC autofocusing system. An initial video image of luminescence was obtained using Hepes buffer containing 3 μg/mL coelenterazine, a lower membrane-permeable substrate of GLase, and a subsequent image was obtained using Hepes buffer containing 3 μg/mL coelenterazine and 50 ng/mL DNP-BSA to determine the effect of antigen stimulation on degranulation.

### Statistical analyses

The unpaired *t*-test was used to compare group differences. The results were considered statistically significant at *p* < 0.05.

## Results

### Enhanced expression of reporter proteins in RBL-2H3 cells using preferred human codon-optimized genes

**Figure 1:**
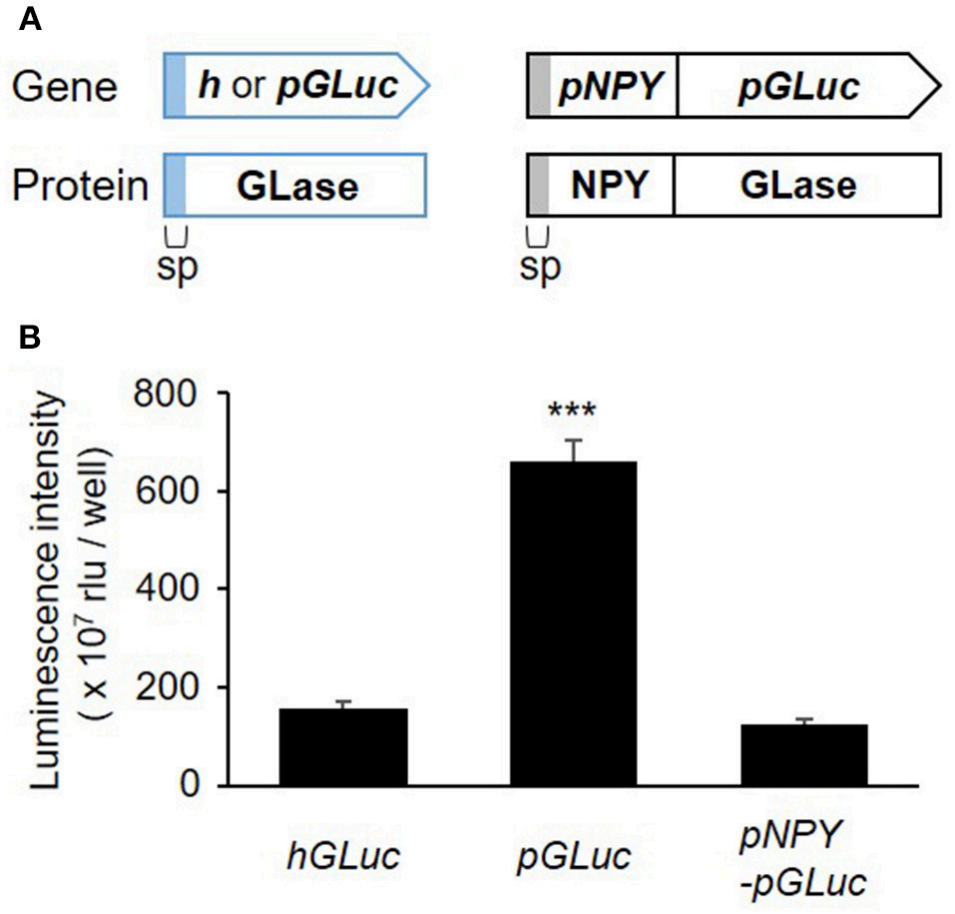
Secretory expression of GLase and NPY-GLase in RBL-2H3 cells. **(A)** Schematic representation of the genes for GLase and NPY-GLase reporter proteins. sp, signal peptide sequence for secretion. **(B)** Luminescence activities of RBL-2H3 cells in the culture medium after 24 h and transfection with the expression vectors pcDNA3-hGLuc (*hGLuc*), pcDNA3-pGLuc (*pGLuc*), and pcDNA3-pNPY-pGLuc (*pNPY*-*pGLuc*). Data represent the means ± SD (*n* = 4, ***p < 0.001 vs *hGLuc*).

recently studies, a simple method for codon optimization by which to express a heterologous protein in mammalian cells has been reported as the “preferred human codon-optimized method” (Inouye et al., 2015; Inouye and Suzuki, 2016). Several bioluminescent proteins, including GLase, were efficiently expressed using the preferred human codon-optimized genes, which were synthesized with preferentially used codons in the human codon table (http://www.kazusa.or.jp/codon/). In our more recent study, we demonstrated that the fusion of proteins proglucagon and GLase was also efficiently expressed in pancreatic α cells using a preferred human codon-optimized gene (Yokawa et al., 2017). As high protein expression of the luciferase protein is advantageous for bioluminescence imaging, we first examined the effect of preferred human codon optimization on the expression of GLase reporter proteins in RBL-2H3 cells. Comparing GLase activities in the culture medium of the RBL-2H3 cells transfected with pcDNA3-pGLuc to that in pcDNA3-hGLuc, pcDNA3-pGLuc exhibited a 4.2-fold higher luminescence activity than pcDNA3-hGLuc. In addition, we found that pcDNA3-pNPY-pGLuc maintained a one-fifth luminescence activity to pcDNA3-pGLuc (Figure 1). These results indicated that the combination of *pNPY* and *pGLuc* was efficient for the expression of NPY-GLase reporter protein in RBL-2H3 cells.

### NPY-GLase as a degranulation reporter protein in RBL-2H3 cells

**Figure 2:**
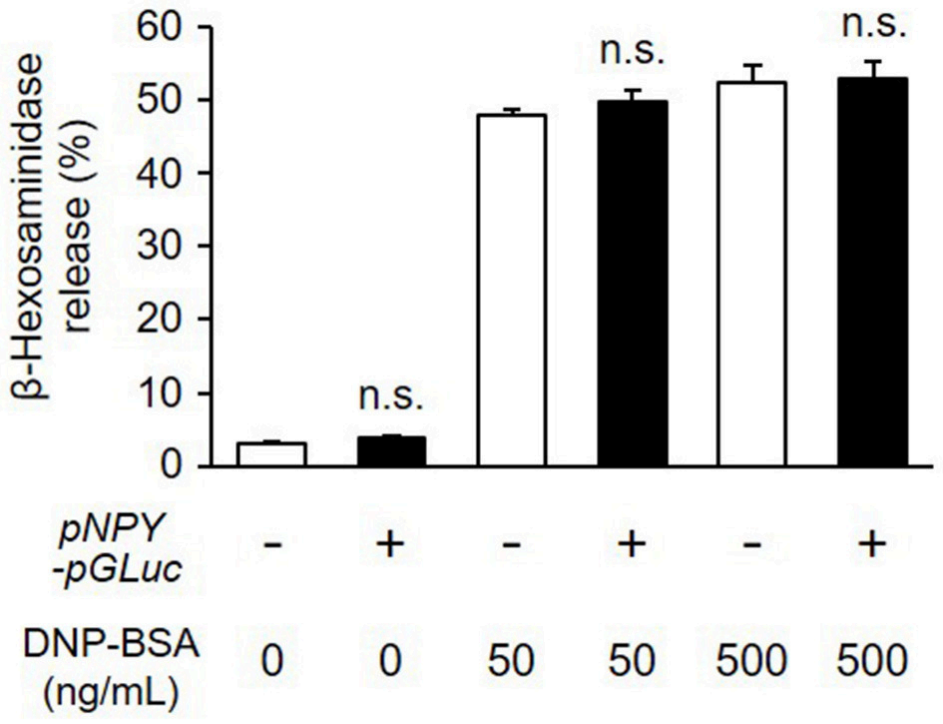
Release of β-hexosaminidase from RBL-NPY-GLase cells by antigen stimulation. Percentages of released β-hexosaminidase to total contents in the cells stimulated with DNP-BSA for 30 min were compared between wild-type cells (open bars) and RBL-NPY-GLase cells (closed bars). Data represent the means ± SD (*n* = 4, n.s. means no significance vs. wild-type).

To study degranulation by bioluminescence imaging, we established a stable transformant of RBL-NPY-GLase cell line expressing NPY-GLase by the transfection with the pcDNA3-pNPY-pGLuc vector. To examine whether the stable expression of NPY-GLase affects degranulation, we compared the amount of β-hexosaminidase released from RBL-NPY-GLase cells to wild-type cells after adding DNP-BSA. The optimal response of wild-type RBL-2H3 cells was induced under our conditions at a dose of 50–500 ng/mL DNP-BSA. The amount of β-hexosaminidase secreted from RBL-2H3 cells expressing NPY-GLase was similar to that in the wild-type cells 30 min after adding DNP-BSA (50 and 500 ng/mL) (Figure 2). This indicated that NPY-GLase expression did not influence degranulation in the RBL-2H3 cells.

**Figure 3:**
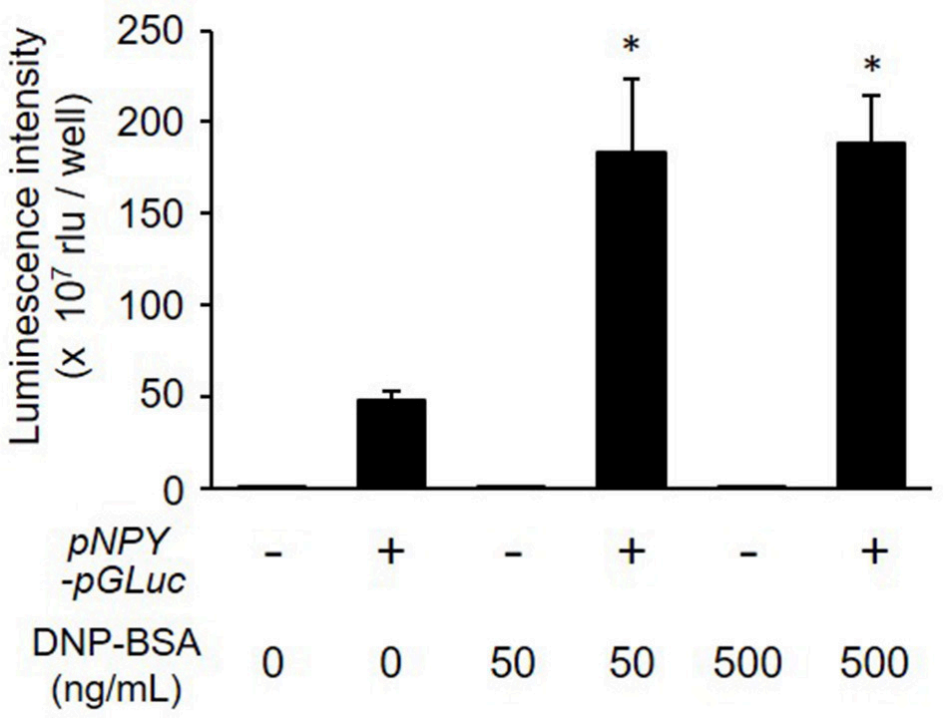
Release of NPY-GLase from RBL-NPY-GLase cells by antigen stimulation. Luminescence activities of NPY-GLase released from the cells stimulated with DNP-BSA for 30 min were compared between wild-type cells (open bars) and RBL-NPY-GLase cells (closed bars). Data represent the means ± SD (*n* = 3, *p < 0.05 vs. without DNP-BSA).

We next examined whether NPY-GLase played a role as a reporter protein in detecting degranulation. The luminescence activity of NPY-GLase secreted into the extracellular medium was detected in antigen-stimulated RBL-2H3 cells stably expressing NPY-GLase but not in the antigen-stimulated wild-type cells (Figure 3). According to β-hexosaminidase secretion, similar bioluminescence activity was measured in RBL-2H3 cells expressing NPY-GLase after adding DNP-BSA (50 and 500 ng/mL). These results suggested that NPY-GLase acts as a degranulation reporter protein in RBL-2H3 cells.

### Bioluminescence imaging of degranulation in RBL-2H3 cells

**Figure 4:**
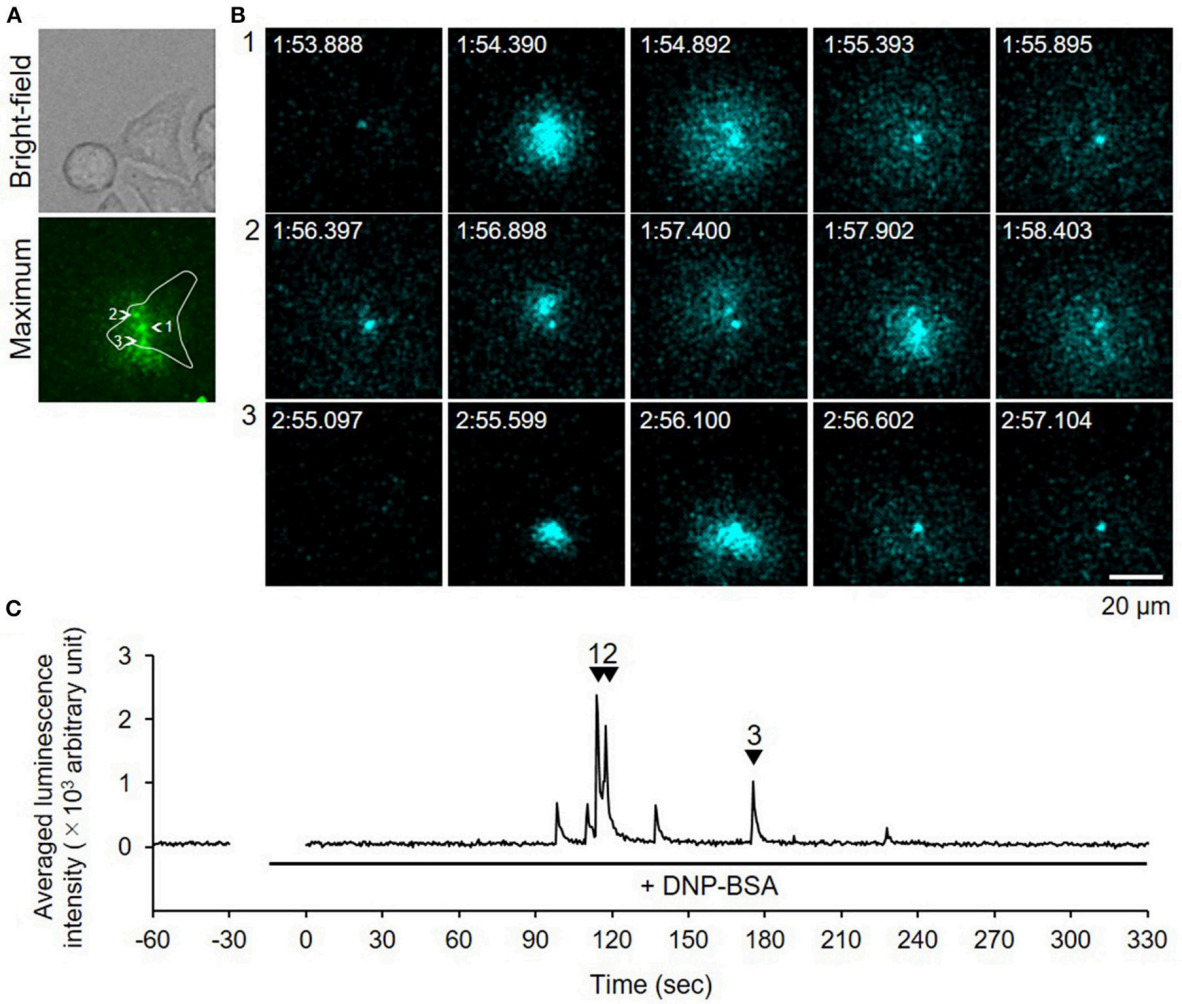
Video-rate bioluminescence microscopy of NPY-GLase secretion from RBL-NPY-GLase cells attached to glass. **(A)** A bright field and luminescence images of RBL-NPY-GLase cells. The focal plane was adjusted to 2 μm from the bottom side of the cells on the glass plate. A “maximum” image shows the maximum luminescence intensity obtained from all frames in the video image after stimulation with DNP-BSA. **(B)** Successive luminescence images of secreted NPY-GLase for a few seconds. Each sequential image (1–3) corresponds to the respective numbers with arrowheads in **C**. **(C)** Time-dependent changes in luminescence intensities of secreted NPY-GLase in the indicated area corresponding to the single cell in the “maximum” image.

We used video-rate bioluminescence microscopy to visualize degranulation from living RBL-NPY-GLase cells by detecting the luminescence signals of NPY-GLase. The cells sensitized with anti-DNP IgE on a 35-mm glass-bottom dish were stimulated with DNP-BSA in Hepes buffer containing coelenterazine. The luminescence video images were recorded at an exposure time of 500 ms/frame. The luminescence signals from the cells were rarely detected, while the luminescence signals from secreted NPY-GLase were repeatedly detected in single cells after ~90–120 sec after adding DNP-BSA (Figure 4, Supplementary Video 1). The luminescence video image showed that NPY-GLase was secreted from different areas of the cell and diffused into the extracellular space.

**Figure 5:**
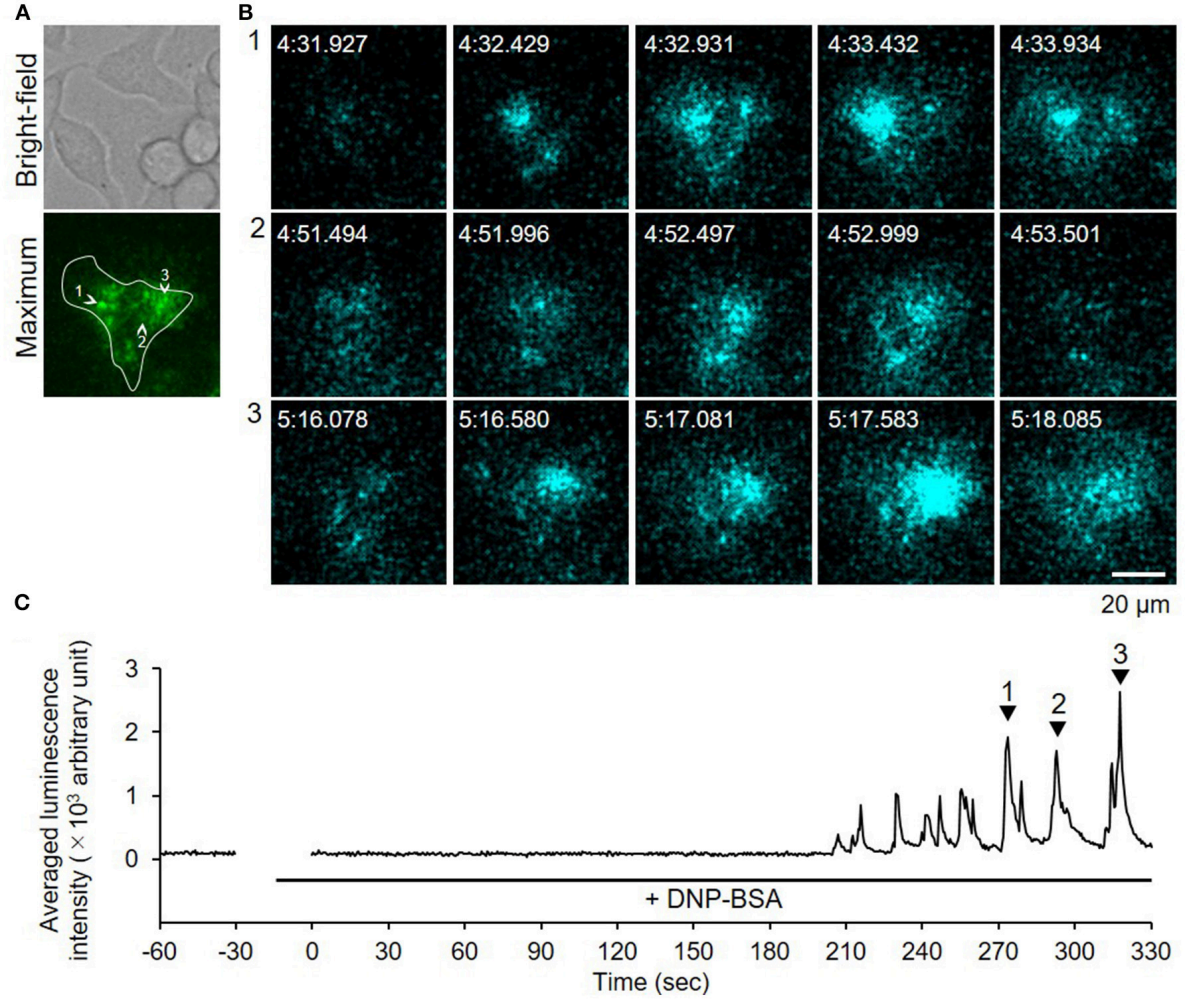
Video-rate bioluminescence microscopy of NPY-GLase secretion from RBL-NPY-GLase cells attached to extracellular matrix ECM gel. **(A)** A bright-field and luminescence images of RBL-NPY-GLase cells. The focal plane was adjusted to 2 μm from the bottom side of the cells on the Matrigel-coated glass plate. A “maximum” image shows the maximum luminescence intensity obtained from all frames in the video image after stimulation with DNP-BSA. **(B)** Successive luminescence images of secreted NPY-GLase for a few seconds. Each sequential image (1–3) corresponds to the respective numbers with arrowheads in **C**. **(C)** Time-dependent changes of luminescence intensities of secreted NPY-GLase in the indicated areas corresponding to the single cell in the “maximum” image.

Because bioluminescence imaging can detect protein secretions from the entire cell surface, the method could be applied to observing degranulation from cells on thick gel (Suzuki et al., 2017). When we measured degranulation of the RBL-2H3 cells on a Matrigel-coated glass-bottom dish after DNP-BSA stimulation, similar luminescence pulse signals were obtained (Figure 5, Supplementary Video 2). This indicated that the bioluminescence imaging technique is a powerful tool by which to visualize degranulation in mast cells adhered to ECM.

## Discussion

The technology of real-time imaging to detect the biological changes in living cells with the development of high-resolution microscopes, specific probes, and various reporter proteins, is crucial for understanding biological events. Exocytosis is the process by which intracellular secretory vesicles are delivered to the plasma membrane and the vesicle contents are released into the extracellular environment (Masedunskas et al., 2012; Tran and Ten Hagen, 2017). This fundamental cellular process is an important biological event, occurs in many types of cells and tissues, and is essential for the release of diverse bioactive molecules that function not only in immunity but also in neurotransmission, the endocrine system, digestion, and reproduction. In recent years, methods using the fluorescence and bioluminescence of specific reporter proteins have expanded to help visualize exocytotic events in real time with high sensitivity. In fluorescence imaging, total internal reflection fluorescence (TIRF) and two-photon excitation fluorescence are the most widely used (Poulter et al., 2015; Takahashi, 2015). Because secretory proteins are transported by secretory vesicles through the ER–Golgi pathway and released to the outside of the cells by exocytosis, TIRF imaging can visualize the movement of these secretory vesicles before exocytosis from the cells within the evanescent field, and two-photon fluorescent imaging can detect the shape of the secretory vesicle during exocytosis; however, these fluorescence methods allow these observations only within limited areas of the cell surface and are not suitable for quantifying the amount of proteins secreted from individual cells.

On the other hand, bioluminescence imaging of protein secretion has been performed based on a luciferin–luciferase reaction, and the amount of secreted protein could be estimated by luminescence intensity. In addition, bioluminescence imaging can visualize protein secretion over the entire cell surface. The secreted luciferase from *G. princeps* (GLase) was found to be the smallest luciferase (168 amino acid residues without a signal peptide sequence) that catalyzes the oxidation of coelenterazine to emit light (488 nm) without any cofactors (Tannous et al., 2005; Inouye and Sahara, 2008; Inouye, 2018). Furthermore, a preferred human codon-optimized GLase gene showed higher luminescence activity than other luciferase genes when expressed in mammalian cells.

In a previous study, to improve time resolution and luminescence sensitivity for detecting luminescence signals, we used an EM-CCD camera attached to a microscope for video-rate bioluminescence imaging using GLase, and visualized the exocytotic secretions of glucagon and insulin from pancreatic α and β cells, respectively, in real time (Suzuki et al., 2007, 2011a; Yokawa et al., 2017). Using our bioluminescence imaging system, we were able to directly observe the exocytotic secretions over 30 min from thick cell spheroids in a 3D culture and from individual cells, and could spatiotemporally estimate the quantitative changes of exocytotic events (Suzuki et al., 2017).

To detect mast cell degranulation at the single living-cell level using fluorescence microscopy, we focused on tetraspanning protein CD63, which is distributed within the intracellular granules and plasma membranes in mast cells. Because the amount of CD63 in the plasma membrane increases according to the amount of degranulation (Furuno et al., 1996), the CD63 expressed on the cell surface was measured using the basophil activation test in present (de Weck et al., 2008; Santos and Lack, 2016). Using the fusion of CD63 and fluorescent proteins in mast cells, we visualized the movement of the intracellular granules and fusion of the granule plasma membranes (Amano et al., 2001; Mori et al., 2002). In fluorescence imaging, the morphological changes based on degranulation were clearly detected, but a quantitative evaluation of degranulation was difficult.

In this study, we established a method of bioluminescence imaging by which to analyze degranulation from single mast cells using the fused protein NPY-GLase because NPY is known to distribute in serotonin-containing secretory granules (Jacobs et al., 2009; Azouz et al., 2012, 2014; Efergan et al., 2016) and GLase is a smallest secretory luciferase that shows high luminescence activity by reacting with coelenterazine in the presence of outside of cells. Since the luminescence intensity corresponds to the amount of GLase released from cells, we considered that it is possible to evaluate the degranulation using this method. When NPY-GLase was efficiently expressed in RBL-2H3 cells after transfection with the expression vector of the preferred human codon-optimized gene (Figure 1), the expressed NPY-GLase did not affect the release of β-hexosaminidase (Figure 2) but enabled us to detect exocytosis as luminescence signals once released outside the cells (Figure 3). Using RBL-2H3 cells expressing NPY-GLase, we succeeded in visualizing degranulation using bioluminescence microscopy based on antigen stimulation and found that NPY-GLase showed a pulsatile release from different areas in a single cell (Figure 4). It is known that mast cells respond to the various size of antigens from soluble hapten-conjugated proteins (~10 nm) used here to hapten-coated beads (~100 μm) (Pierini et al., 1996). The bioluminescence imaging might be useful to visualize the polarized degranulation on the whole surface of mast cells binding to various size of antigens.

Moreover, bioluminescence microscopy made it possible to observe degranulation from cells adhered to matrices despite their thickness (Figure 5). As mast cells are distributed within a variety of bodily tissues and organs, they can attach to distinct adhesion proteins on ECM within a wide range of ECM stiffness. Matrix stiffness is an important factor by which to regulate cell functions, such as proliferation, migration, activation, and differentiation (Discher et al., 2005; Hytönen and Wehrle-Haller, 2016). Bioluminescence imaging is certainly a powerful tool for providing new dynamic information on degranulation, and is applicable to investigations on the relationship between adhesion to ECM proteins and mast cell activation using plate-coating ECM proteins on matrices of varying stiffness.

## Author contributions

SY and AH performed the experiments, assembled the figures, and analyzed the data. TS and SI developed the instruments and interpreted the data. YI assisted in data analysis and interpretation, and TF designed the study and wrote the paper.

### Conflict of interest statement

SI is employed by JNC Corporation. The remaining authors declare that the research was conducted in the absence of any commercial or financial relationships that could be construed as a potential conflict of interest.

## Supplementary material

Supplementary Figure 1 Expression vectors for NPY-GLase. **(A)** Nucleotide sequence of preferred human codon-optimized gene of NPY with the signal peptide sequence used in the expression vectors for NPY-GLase. Amino acids corresponding to nucleotides are also presented. **(B)** Construction of the expression vectors for NPY-GLase. To express the fusion of NPY protein to the N-terminus of GLase, the expression vector pcDNA3-pGLuc-pN was used (Yokawa et al., 2017). The *pNPY* fragment was inserted into pcDNA3-pGLuc-pN to obtain and pcDNA3-pNPY-pGLuc. **(C)** Nucleotide sequence between pNPY and pGLuc.

Supplementary Video 1 Luminescence image of NPY-GLase secretion from an RBL-NPY-GLase cell attached to glass. The video image of secreted NPY-GLase corresponds to Figure 4. Luminescence signals of the secreted NPY-GLase according to DNP-BSA stimulation were recorded at an exposure time of 500 ms and reading time of 1.712 ms per image. The luminescence signals (cyan) were superimposed on the bright-field image. The movie plays at 10× speed.

Supplementary Video 2 Luminescence image of NPY-GLase secretion from an RBL-NPY-GLase cell attached to ECM gel. The video image of secreted NPY-GLase corresponds to Figure 5. Luminescence signals of the secreted NPY-GLase according to DNP-BSA stimulation were recorded at an exposure time of 500 ms and reading time of 1.712 ms per image. The luminescence signals (cyan) were superimposed on the bright-field image. The movie plays at 10× speed.
