# Supplementary material for: Video-Rate Bioluminescence Imaging of Degranulation of Mast Cells Attached to the Extracellular Matrix
Source: Front Cell Dev Biol. 2018 Jul 10;6:74. doi: 10.3389/fcell.2018.00074 (PMC6048188; doi:10.3389/fcell.2018.00074)
Supplement: Supplementary Figure 1 — Expression vectors for NPY-GLase. (A) Nucleotide sequence of preferred human codon-optimized gene of NPY with the signal peptide sequence used in the expression vectors for NPY-GLase. Amino acids corresponding to nucleotides are also presented. (B) Construction of the expression vectors for NPY-GLase. To express the fusion of NPY protein to the N-terminus of GLase, the expression vector pcDNA3-pGLuc-pN was used (Yokawa et al., 2017). The pNPY fragment was inserted into pcDNA3-pGLuc-pN to obtain and pcDNA3-pNPY-pGLuc. (C) Nucleotide sequence between pNPY and pGLuc. [file Presentation_1.PPTX]

## Slide 1
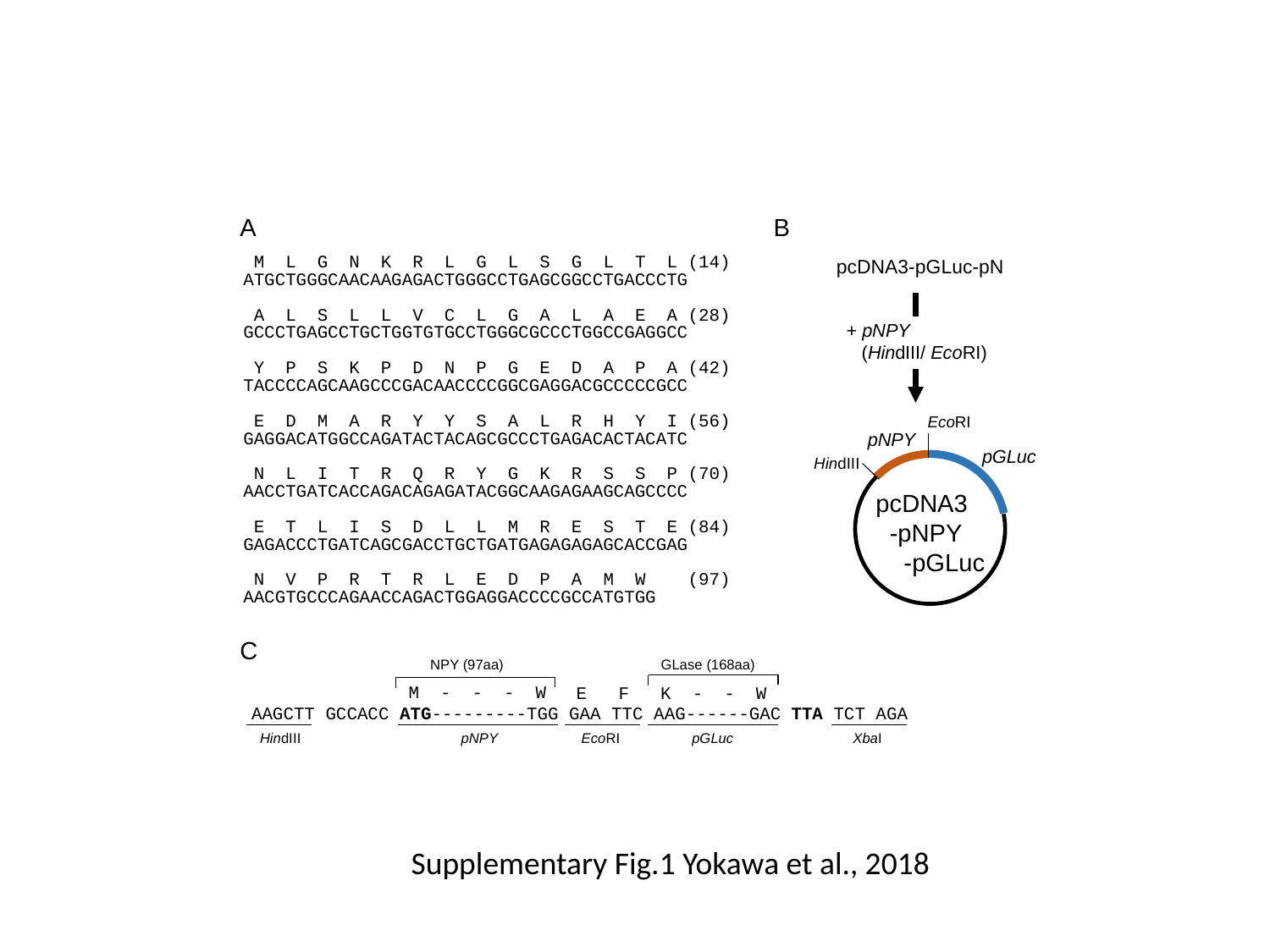

B
A
 M L G N K R L G L S G L T L (14)
 ATGCTGGGCAACAAGAGACTGGGCCTGAGCGGCCTGACCCTG
 A L S L L V C L G A L A E A (28)
 GCCCTGAGCCTGCTGGTGTGCCTGGGCGCCCTGGCCGAGGCC
 Y P S K P D N P G E D A P A (42)
 TACCCCAGCAAGCCCGACAACCCCGGCGAGGACGCCCCCGCC
 E D M A R Y Y S A L R H Y I (56)
 GAGGACATGGCCAGATACTACAGCGCCCTGAGACACTACATC
 N L I T R Q R Y G K R S S P (70)
 AACCTGATCACCAGACAGAGATACGGCAAGAGAAGCAGCCCC
 E T L I S D L L M R E  S T E (84)
 GAGACCCTGATCAGCGACCTGCTGATGAGAGAGAGCACCGAG
 N V P R T R L E D P A M W (97)
 AACGTGCCCAGAACCAGACTGGAGGACCCCGCCATGTGG
pcDNA3-pGLuc-pN
+ pNPY
 (HindIII/ EcoRI)
EcoRI
pNPY
pGLuc
HindIII
pcDNA3
 -pNPY
 -pGLuc
C
GLase (168aa)
NPY (97aa)
M - - - W
E F
K - - W
 AAGCTT GCCACC ATG---------TGG GAA TTC AAG------GAC TTA TCT AGA
HindIII
pNPY
EcoRI
pGLuc
XbaI
Supplementary Fig.1 Yokawa et al., 2018
